# Pyoderma gangrenosum and sickle cell vaso-occlusive crisis: fortuitous association or causal relationship ?: a case report

**DOI:** 10.1186/s13256-026-06185-7

**Published:** 2026-08-09

**Authors:** Abderamane Yacoub Hadje Kaltam, Moussa Seck, Elimane Seydi Bousso, Khadim Diop, Alioune Badara Diallo, Fatma Dieng Bousso, Blaise Felix Faye, Eric Loic Bana, Hadje Zara Issa, Saliou Diop

**Affiliations:** 1 Department of Clinical Hematology, National Blood Transfusion Center, Cheikh Anta Diop University of Dakar, Dakar, Sénégal; 2 Medical Oncology and Clinical Hematology department, Principal Hospital of Dakar, Dakar, Sénégal; 3 Dermatology department, Albert Royer National Children’s Hospital of Dakar, Dakar, Sénégal

**Keywords:** Pyoderma gangrenosum, Sickle cell disease, VOC, Corticosteroid therapy

## Abstract

**Background:**

The association between pyoderma gangrenosum (PG) and painful vaso-occlusive crisis (VOC) in sickle cell disease (SCD) patients is a rare clinical entity, rarely reported in the literature (it is the 3rd published case). It illustrates a critical therapeutic dilemma: corticosteroids, the standard treatment for PG, exacerbate the vaso-occlusive crises of sickle cell disease. Its interest is also educational, aiming to alert clinicians to this association. The novelty lies in the description of a complex and successful management of a PG flare-up during an active vaso-occlusive crisis, a poorly documented scenario. This case thus provides a practical model for managing this therapeutic paradox and paves the way for considering a potential shared inflammatory pathophysiology between the two diseases.

**Case presentation:**

A 23-year-old female of Senegalese origin presented with persistent, hyperalgesic VOC involving the coastal region and limb pain. The symptom progression was marked by the appearance of sero-hemorrhagic bullous lesions, which evolved into irregular-bordered ulceronecrotic lesions affecting the limbs (anterior thighs). The patient’s history revealed similar episodes over the past seven years (since 2017). Bacteriological examination of the fluid was sterile. Consequently, a diagnosis of pyoderma gangrenosum (PG) was made. Treatment with corticosteroids, combined with local wound care and monthly transfusion exchanges, led to favorable outcomes.

**Conclusion:**

Managing pyoderma gangrenosum (PG) in patients with sickle cell disease (SCD) is challenging because corticosteroid therapy, the treatment of choice for PG, often triggers vaso-occlusive crises (VOCs). Therefore, recognizing this association is crucial.

## Introduction

Pyoderma gangrenosum (PG) is a rare inflammatory dermatosis clinically characterized by rapidly progressing, extremely painful cutaneous ulcers with undermined, irregular, erythematous-violaceous borders, and mucopurulent or hemorrhagic exudate [1–3]. Several clinical variants have been described, including ulcerative, bullous, pustular, vegetative, peristomal, and classic postoperative forms [3].

PG is associated with systemic diseases in 50–70% of cases, such as hematological disorders, inflammatory bowel disease (IBD), and rheumatoid arthritis [4]. However, the association between PG and sickle cell disease (SCD) has been rarely reported; to our knowledge, only two cases have been documented in the literature [5, 6]. A vaso-occlusive crisis (VOC) is an acute complication of SCD that can be precipitated by infections, fever, dehydration, decreased pH, cold, humidity, and certain treatments like corticosteroids [7, 8].

We report a case of PG occurring during a sickle cell VOC at the Clinical Hematology Department of the National Blood Transfusion Center in Dakar, Senegal. The question arises whether this is a fortuitous association or a causal relationship.

## Case presentation

**Fig. 1:**
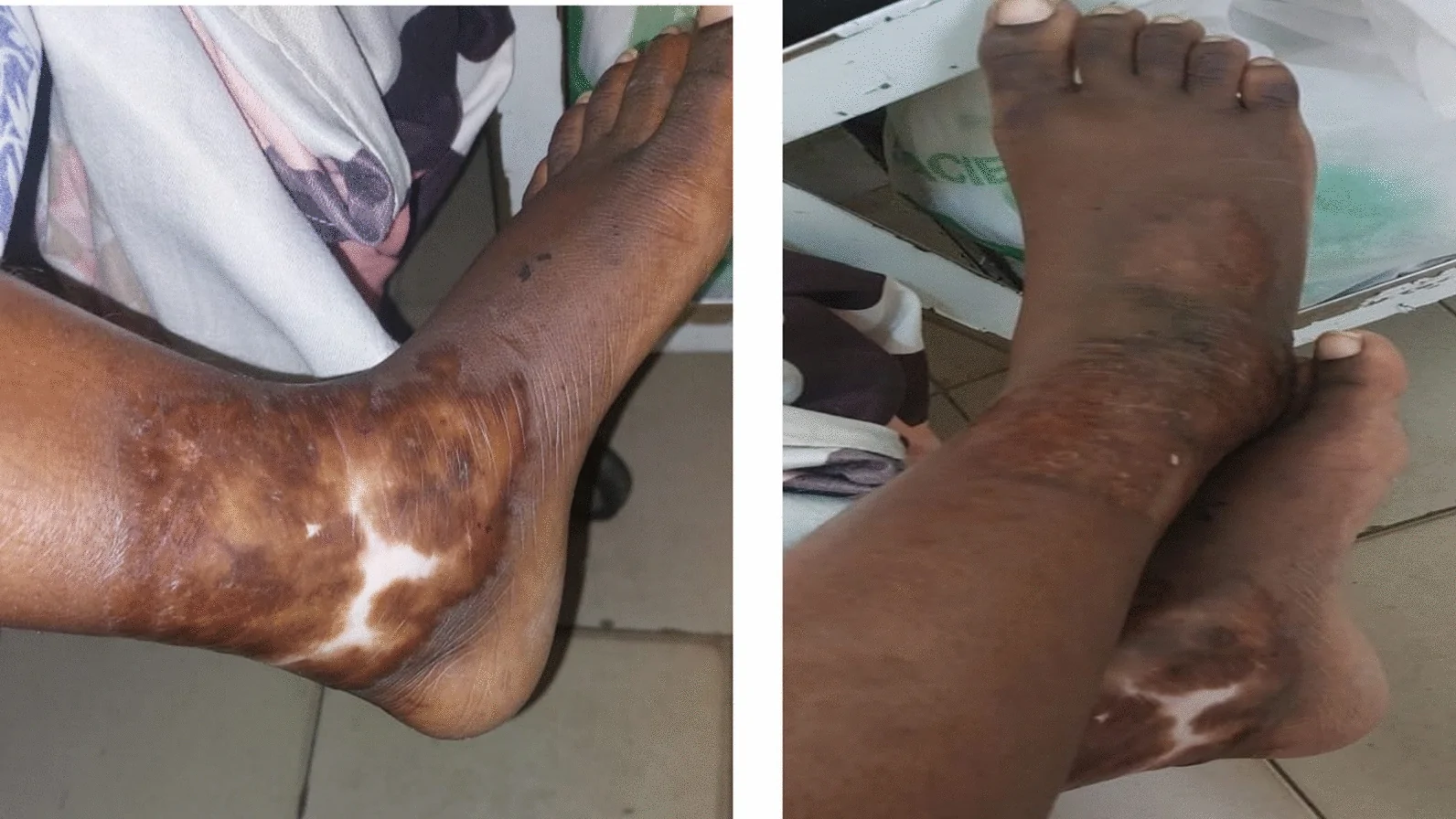
Scarred ulcer-like lesions observed for 6 years

We present the case of a 23-year-old female patient of Senegalese origin who had been followed since 2017 for sickle cell disease (SCD), specifically a compound heterozygote form (SC genotype). She experienced fewer than three vaso-occlusive crises (VOCs) per year, with no chronic complications, and was on daily folic acid supplementation (5 mg/day) and on-demand analgesics. She was hospitalized for persistent, hyperalgesic VOCs, with a Visual Analog Scale (VAS) score of 9/10. On admission, she reported a medical history of pyoderma gangrenosum (PG) in 2017, which responded well to corticosteroid therapy, leaving ulcer scars on her feet (Fig. 1).

Physical examination revealed a hemodynamically poorly tolerated anemic syndrome without external bleeding, respiratory distress with ambient air desaturation at 89%, bilateral pulmonary condensation syndrome, and bowel habit disturbance consisting of semi-liquid diarrhea associated with abdominal pain and vomiting. Paraclinical investigations showed hyperleukocytosis at 16.9 G/L, hypochromic microcytic anemia with hemoglobin at 9.4 g/dL, and platelets at 468 G/L. C-reactive protein (CRP) was elevated at 48 mg/L. A chest X-ray was normal, and abdominal ultrasound revealed moderate free ascites associated with increased portal vein trunk velocity without signs of portal hypertension. The diagnosis of costal VOC triggered by common bacterial pneumonia associated with acute enteritis was retained. The patient received dual antibiotic therapy, Step I and III analgesics, hyperhydration, and oxygen therapy.

**Fig. 2:**
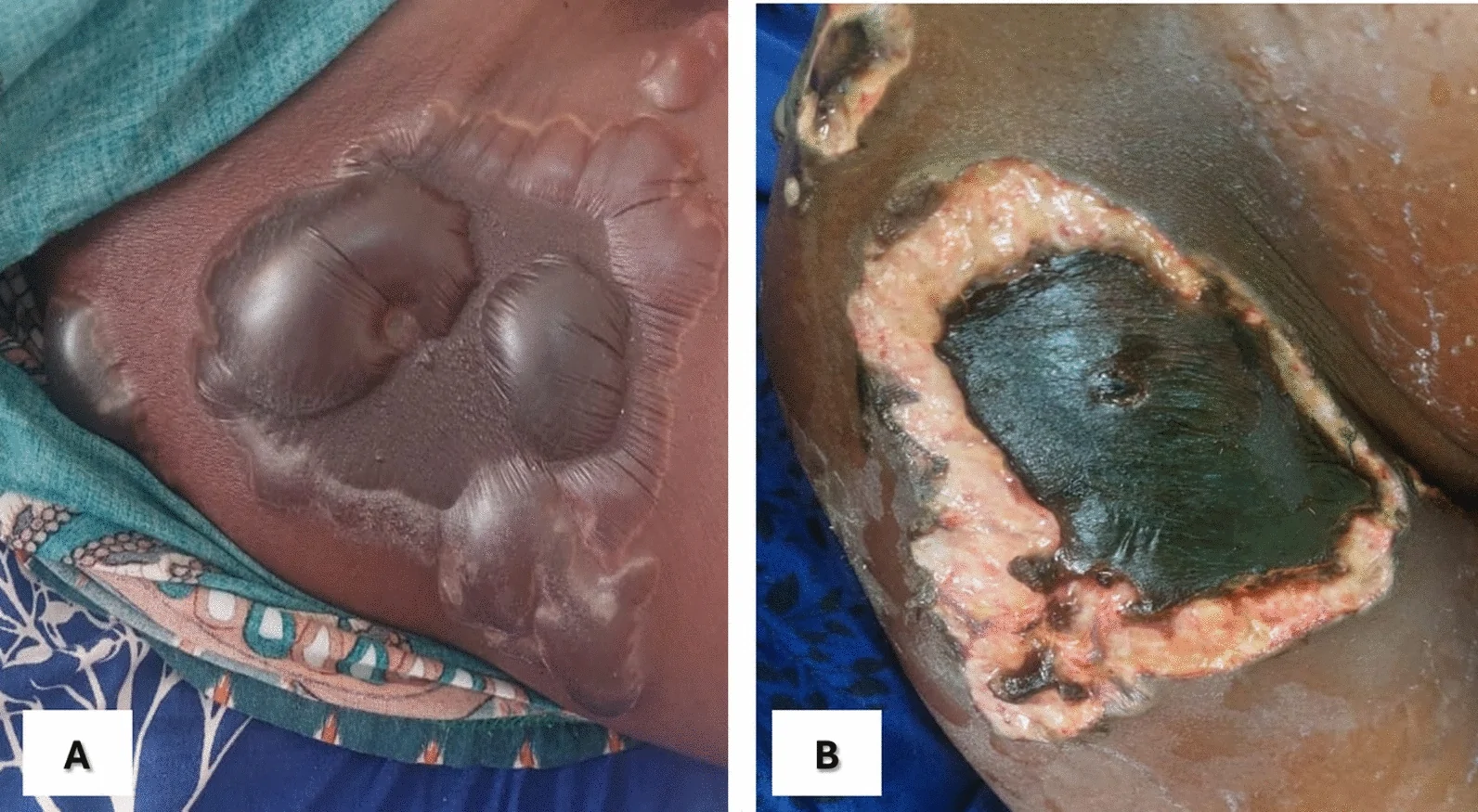
(initial presentation at T = 0): Bullous lesions (**A**) at onset and ulceronecrotic lesions (**B**) after 5 days of evolution on the anterolateral aspect of the right thigh

**Fig. 3:**
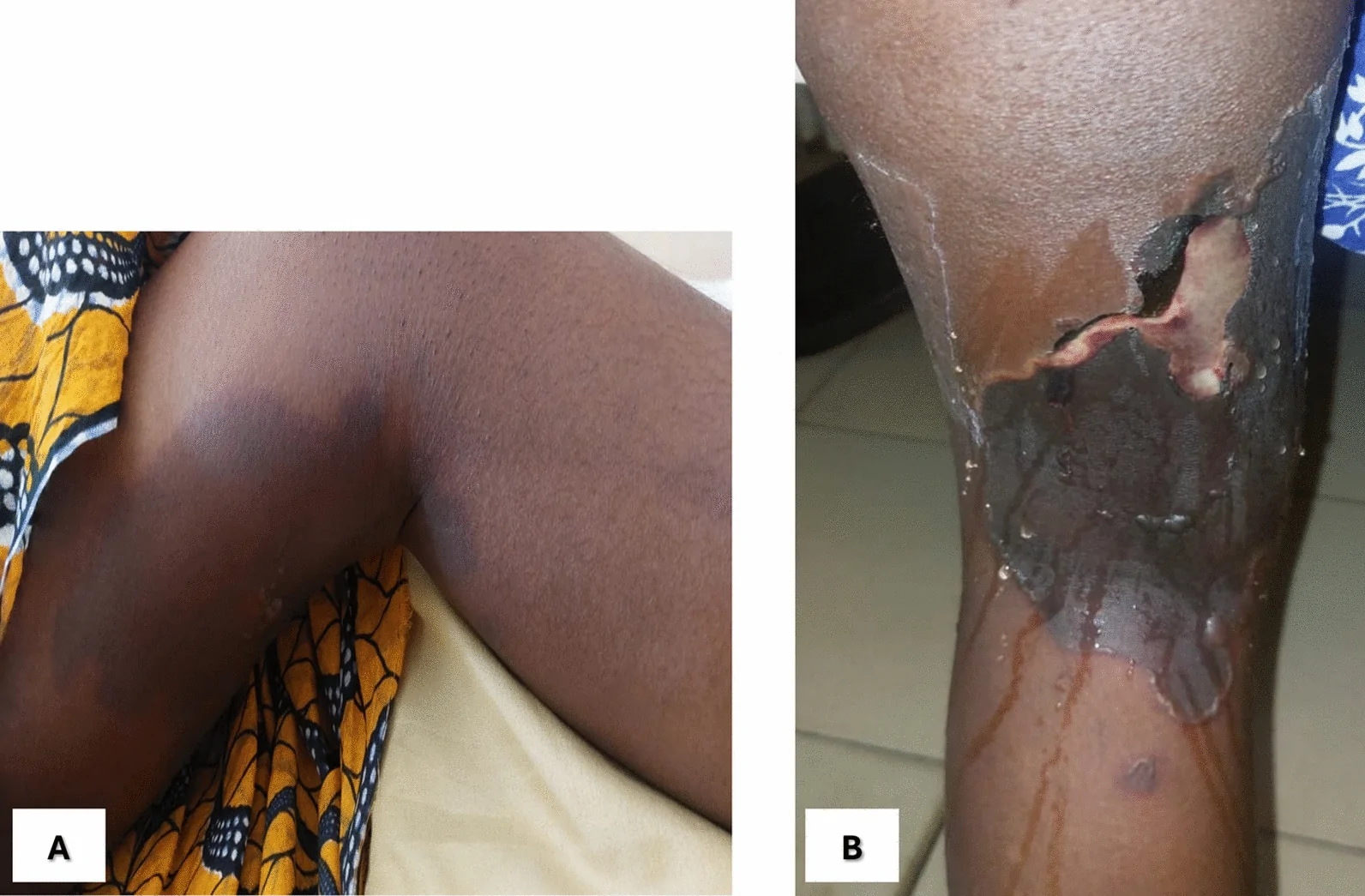
(initial presentation at T = 0): Erythematous lesion (**A**) at onset and ulceronecrotic lesion (**B**) after 6 days of evolution on the posterior aspect of the left knee

**Fig. 4:**
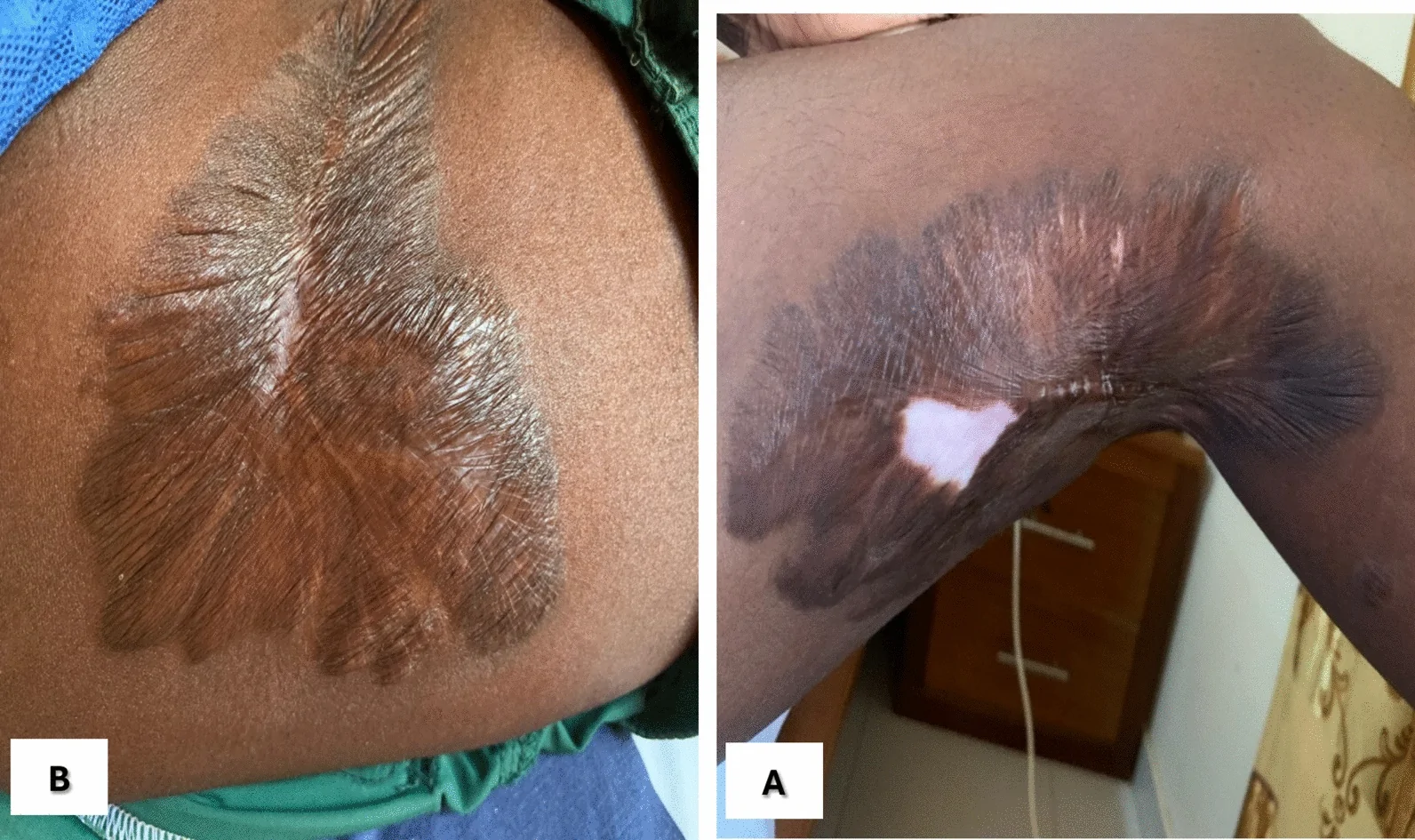
(follow-up at T = 1 month): Dystrophic scarred lesions on the posterior aspect of the left knee (**A**) and the anterolateral aspect of the right thigh (**B**) after 1 year of treatment

After six days, the course was marked by the appearance of bullous lesions, initially blisters with serohematic content (Fig. 2, A and B), which progressed to ulceronecrotic lesions with irregular borders (Fig. 3, B). These lesions affected the lower limbs (anterior thighs), upper limbs (anterior forearms), and buttocks. A diagnosis of recurrent PG was considered and confirmed. Biologically, the culture of the cutaneous serohematic fluid was negative for microorganisms. She received daily local Dakin dressings after blister rupture, triple antibiotic therapy (third-generation cephalosporin, metronidazole, and aminoglycoside), and a bolus of solumedrol 10 mg/kg, followed by a maintenance dose of 1.5 mg/kg. Maintenance treatment with prednisone at 1 mg/kg/day (60 mg/day) was initiated, with progressive tapering over 5 months to a maintenance dose of 5 mg/day, combined with local Dakin care and monthly transfusion exchanges. The patients improved favorably under treatment. Long-term follow-up showed complete healing of the skin lesions, which became dystrophic (Fig. 4).

## Discussion

Pyoderma gangrenosum (PG) is an inflammatory neutrophilic dermatosis characterized by recurrent cutaneous ulcerations with mucopurulent or hemorrhagic exudate [4]. Precise epidemiological data on PG are lacking. The peak incidence occurs between 20 and 50 years of age, with women more frequently affected than men [3, 9]. The clinical features, notably sterile cutaneous ulcerations, female predominance, and age of onset, are also consistent with our observation.

The pathogenesis of PG is complex and involves profound dysregulation of innate and adaptive immune components, with the follicular unit increasingly recognized as the presumed initial target [10]. Following antigenic priming in predisposed individuals, a T helper (Th)17/Th1 skewing leads to a self-perpetuating, neutrophil-dominant auto-inflammatory environment with elevated levels of tumor necrosis factor-alpha (TNF-α), interleukin (IL)-1β, IL-1α, IL-8, IL-12, IL-15, IL-17, IL-23, and IL-36 [11–13]. Pathogenic variants of genes involved in inflammasome formation, including PSTPIP1, MEFV, NLRP3, NLRP12, and NOD2, leading to exaggerated IL-1β release, have been documented in syndromic (for example, PAPA) and sporadic cases of PG [14, 15]. Trauma is a well-documented triggers for PG, involving the release of PG-driving cytokines such as IL-36 and IL-8 from keratinocytes, sufficient in context of genetic predisposition [3]. The complement system, particularly anaphylatoxin C5, NETosis, regulatory T-cell imbalance, B lymphocytes, fibroblasts and monocytes/macrophages, contribute to the multilayered PG pathophysiology [16–19].

PG can be idiopathic or associated with systemic conditions such as inflammatory bowel disease, rheumatological disorders like rheumatoid arthritis, or auto-inflammatory syndromes such as PAPA (pyogenic arthritis, pyoderma gangrenosum, and acne) and SAPHO (synovitis, acne, pustulosis, hyperostosis, osteitis) [5, 20–22]. It is most frequently found in hematological malignancies like multiple myeloma, leukemias, and myeloproliferative syndromes [21, 23]. However, the association with sickle cell disease (SCD) has only been reported in two cases [5, 6]. Our patient represents the third reported case of PG associated with SCD.

The diagnosis was established based on Delphi consensus criteria: exclusion of infection (negative skin culture), history of pyoderma, peripheral erythema, undermined border, tenderness at the ulcer sites, multiple ulcerations, and cribriform scarring at healed sites. However, a skin biopsy was not performed [2].

Optimal treatment involves systemic corticosteroids and cyclosporine A. Steroids combined with cytotoxic drugs are used in resistant cases. Steroids with sulfonamides or immunosuppressants serve as steroid-sparing options. Inconclusive studies and lack of validated diagnostic and response criteria hinder new treatment discovery for PG [4, 24].

Although effective, corticosteroid therapy in this case exacerbated vaso-occlusive crises (VOCs). The literature reports unfavorable PG course in SCD context, including death due to chest pain [5]. Still, transfusion exchanges, pain management, and rehydration led to satisfactory response in our patient.

This rare association and VOC exacerbation may be due to under-recognition of this entity or other common causes like corticosteroid therapy. PG pathophysiology remains incompletely understood, especially in SCD context [3, 15].

## Conclusion

This case demonstrates that treating pyoderma gangrenosum (PG) in sickle cell disease (SCD) patients is challenging because corticosteroid therapy the standard PG treatment, exacerbates vaso-occlusive crises (VOCs) potentially compromising patient prognosis. Alongside medical treatment, appropriate wound care, pain management, and VOC prevention through transfusion exchanges are essential in managing PG cases in SCD patients.

## Data Availability

The datasets used and/or analysed during the current study are available from the corresponding author on reasonable request.
